# Regional Position and Axonal Environment Shape Astrocyte Morphology in the Mouse Optic Projection

**DOI:** 10.1007/s12035-025-05552-7

**Published:** 2025-11-28

**Authors:** Joseph Matthew Holden, David John Calkins

**Affiliations:** Department of Ophthalmology and Visual Sciences, Vanderbilt University Medical Center, Nashville, TN 37212 USA

**Keywords:** Astrocyte, Morphology, Optic nerve, Chiasm

## Abstract

Optic neuropathies encompass a diverse group of disorders that result in damage to the optic nerve and lead to vision loss. Despite the initial site of damage often being injury specific, we have an incomplete understanding of how differential regional susceptibility arises. Recent studies have implicated astrocytes in this phenomenon in mice, showing regional transcriptional heterogeneity in optic nerve astrocytes in response to glaucoma and optic nerve crush. Because astrocytes provide structural support and protection from tissue deformation, we examined the length of the optic nerve for astrocyte morphology differences which could alter its biomechanical properties, and by extension, susceptibility to injury. We report that astrocyte morphology changes along the length of the nerve, with average process angle smoothly rotating in close accordance with a changing tissue environment outside the nerve. Moreover, astrocyte morphology appears driven by the local axonal environment, with cells in the chiasm more closely resembling astrocytes in the brain rather than the rest of the optic nerve or tract- likely due to the complex axon orientation that arises there. Astrocytes in the nerve also exhibit the same neuronal-associated microstructures we identified in our previous work in the retina. These observations open the possibility that astrocytes participate in a feedback loop where their structure responds to local conditions, and in turn, shapes the tissue’s biomechanical properties- modulating its vulnerability to injury which may itself drive changes in the surrounding milieu.

**Supplementary Information:**

The online version contains supplementary material available at 10.1007/s12035-025-05552-7.

## Introduction

Optic neuropathies are disorders which damage the optic nerve, often leading to vision loss. Damage can arise from a variety of sources, including underlying disease processes or from physical insults like tumor growth or trauma. Damage can occur throughout the nerve, and initial localization is often injury specific. For example, in glaucoma, an early site of stress is the optic nerve head (ONH)[1]. In contrast, pituitary adenomas can exert a compressive force at the optic chiasm, primarily damaging nasal retinal ganglion cell axons as they cross to the contralateral projection[2]. Moreover, traumatic injuries to the forehead or temporal skull often damage the region of the nerve within the optic canal, where it adheres to the bone through the dura[3]. Varied patterns of injury highlight the complexity of optic nerve pathology and underscore the need for deeper understanding of the progression and regional susceptibility of the optic nerve to damage at a cellular level.

Astrocytes, a major class of macroglial cells in the optic nerve, likely play a significant role in susceptibility to tissue pathology. In addition to their functions to maintain tissue homeostasis, astrocytes serve as a compliant embedding for neurons, absorbing force in the case of mechanical deformation[4]. This is relevant in glaucoma where intraocular pressure (IOP) exerts a posterior-directed force at the optic nerve head and in traumatic injuries which can exert tensile, compressive and rotational forces during impact[5, 6]. Moreover, optic nerve tissue adaptations to injury are largely mediated by astrocytes, which respond by attempting to normalize tissue homeostasis[7]. Throughout the CNS, glial responses to injury vary significantly on a spectrum of those having a protective effect to maladaptive changes which exacerbate degeneration. Understanding what influences a particular astrocyte to engage a given program of reactivity is important when attempting therapeutic interventions.

One high level determinant of reactivity program choice is cellular localization within a tissue. Recent studies have shown spatially distinct transcriptional heterogeneity in optic nerve astrocytes, both in naïve mice and also in response to insult[8–10]. These studies show a distinct functional divergence between astrocytes in the unmyelinated optic nerve head and the myelinated optic nerve proper. Whether there is further specialization at regional levels within each compartment is an important, yet open question.

When assessing glial reactivity, it is essential to consider morphological data[11]. It is possible that high dimension morphological data encodes structural features indicative of classes of astrocytes which may respond in distinct ways to insult. Towards this aim, we recently used a mouse line (G-MORF) that stochastically labels cells expressing glial fibrillary acidic protein (Gfap) with a membrane-directed, highly antigenic fluorescent protein. These mice allow for high throughput analysis of astrocyte morphology at single-cell resolution. We used these mice to characterize astrocytes in the retina for both naïve and hyperglycemic animals[12–14]. In that work, we found that the surrounding neuronal and vascular architecture were highly correlated with morphological features of the retinal astrocytes. Similarly, others have characterized aspects of astrocyte morphology in the optic nerve. Single cell dye filling experiments show three morphological classes of astrocytes in both the rat and mouse optic nerve[15–17]. These classes include longitudinally, transversely, and randomly oriented astrocytes. A few studies also compare single cell astrocyte morphology in the unmyelinated region of the optic nerve to the myelinated nerve as a whole[18–20]. In our previous studies in the retina, we found that G-MORF astrocyte morphology revealed more detail than from transgenic animals which localize fluorescent protein to the cytoplasm. Additionally, G-MORF astrocyte labeling often shows improved morphology in the retina compared to dye filling, as sometimes cells fill incompletely.

Here, we expand on these studies, characterizing astrocyte morphology along the length of the myelinated optic nerve and extend analysis into the optic chiasm and optic tract. In this study, we wanted to see (1) if G-MORF mice reveal any astrocyte structural elements that were not observed in prior optic nerve studies which used dye filling or cytosolic fluorescent markers, (2) determine if astrocyte morphology varies along the length of the myelinated optic nerve, and (3) characterize optic chiasm and optic tract astrocyte morphology. We report similar morphological detail to previous studies but connect observations in optic nerve to those observed in the retina and extend analysis into the chiasm and optic tract[12]. Additionally, we find the net polarization of astrocyte morphology changes smoothly along the length of the nerve, and overall cell shape correlates with the axonal environment. These data add to our understanding of optic nerve astrocyte morphology and open the possibility that there are biomechanical differences along the length of the nerve which may be relevant to injury susceptibility.

## Methods

### Animals

All animals used in this study were adult mice (2–3 months) on a C57 Black 6 background, with equal numbers of males and females. Animals were housed at the Vanderbilt University Division of Animal Care facility and subjected to a 12-h light/dark cycle. Animals were provided with water and rodent chow ad libitum. Cre 77.6 mice (Jackson Labs #024098) were crossed with MORF3 mice (Jackson Labs #035403) to generate G-MORF mice. Animals were deeply anesthetized using pentobarbital until the toe pinch reflex was abolished. The thoracic cavity was then opened, and animals were perfused with a solution of phosphate buffered saline (PBS) followed by 4% paraformaldehyde in PBS.

### Histology

Frozen longitudinal sections were acquired on a Thermo Fisher CryoStar NX50 at a thickness of 25–30 µm. Coronal paraffin sections were acquired at a thickness of 15–25 µm. For coronal sections, images of paraffin sections were taken on an iPhone 16 prior to deparaffination (Fig. S1). The angle of the paraffin section relative to the slide edge was noted from these images and used to correct measurements of astrocyte process angle. Both paraffin and frozen section immunohistochemistry was performed as previously described [12, 21]. Primary antibodies used include: rabbit anti-V5 (1:500, Bethyl Laboratories, A190-120A), goat anti-V5 (1:500, Abcam, ab95038), goat anti-Gfap (1:500, Abcam, ab53554), mouse anti-Gfap (1:500, Millipore, MAB360), mouse anti-SMI-31 (1:500, Biolegend, 801,601), and mouse anti-beta III tubulin (1:500, Millipore, MAB5564).

Secondary antibodies used in this paper include donkey anti-rabbit conjugated to Alexa 488 (1:200, Jackson ImmunoResearch, 711–545–152), donkey anti-goat conjugated to Alexa 488 (1:200, Jackson ImmunoResearch, 705–545–003), donkey anti-rabbit conjugated to Alexa 555 (1:200, Invitrogen, A-31572), donkey anti-goat conjugated to Cy3 (1:200, Jackson ImmunoResearch, 705–165–147), donkey anti-mouse conjugated to Alexa 488 (1:200, Jackson ImmunoResearch 715–545-151) and donkey anti-mouse conjugated to Alexa 555 (1:200, Jackson ImmunoResearch 715–565-151). Images are taken in grayscale and pseudo-colored in figures.

### Imaging and Analysis

Images were taken on a Nikon Spinning Disk confocal microscope at magnifications of 20X and 60X. Coronal sections labeled for Gfap were imaged in a Z-stack at 20X and collapsed into a single image using the standard deviation stacking method in Fiji ImageJ. The CLAHE enhance local contrast function was then applied. To determine average angle of Gfap processes, the Fiji ImageJ “Ridge Detection” algorithm was run with the following parameters: line_width = 6, high_contrast = 255, low_contrast = 10, sigma = 1.2, lower_threshold = 0, upper_threshold = 3.74, minimum_line_length = 2, maximum = 200. The resulting CSV and ROI files were read into a Python script to find the start and end position of each ridge contour. The angle made between the line from the start and end position and the horizontal axis of the image were recorded. Each angle measurement was weighted by the length of the ridge line and the average was then taken. This average angle was corrected by the angle made between the paraffin sections and the slide edge prior to deparaffination to correct for deviations which occur simply due to hand placement of sections on each slide (*see above*). Angle measurements were plotted as a function of optic nerve position and the points were fit using 7th degree polynomials using the Python package Scikit-learn. Critical points were found using the Python library SymPy.

Independently, optic nerve tissue environment was analyzed using the image dataset from *Foo *et al. [22] Optic nerve location was manually assessed from 10 µm thick cryosection color images. In each section where the optic nerve or chiasm were visible, we noted the tissue identity surrounding the optic nerve and chiasm. This includes the hypothalamus and diagonal band nucleus of the brain, the ventral aspect of the presphenoid bone and the optic foramen, the trigeminal nerve, extraocular rectus muscles, lacrimal and Harderian glands, and sclera of the globe. This was accomplished using the Allen Brain Atlas, a whole-body mouse computed tomography (CT) scan atlas by IMAIOS (https://www.imaios.com/en/vet-anatomy/mouse/mouse-whole-body), and a variety of publications[23, 24]. When a change in the combination of surrounding tissues occurred, we noted the location. These locations were mapped to an optic nerve of length 1 for normalization that would facilitate comparison to our own data. The absolute value of the difference between these locations and the inflection points in Gfap labeling were computed.

Longitudinal and transverse/vertical Gfap process segments from paraffin-sectioned nerves were found using the Fiji ImageJ particle analysis tool. Images were first binarized using the “Auto Local Threshold” tool using the Bernsen method with a radius of 3. Particles found with area 0–25µm^2^ were counted as longitudinal processes and larger particles were counted as transverse or vertical. We found this to be an optimal cutoff based off the dimensions of individually traced processes throughout the tissue. ROIs for each group were subject to XOR operation and the total area acquired using the Measure function in Fiji ImageJ. The XOR operation simply combines all the ROIs into a single complex polygon who’s area can be easily found in one operation. Axon orientation and astrocyte process orientation were compared for 60X optic nerve images from each optic nerve region (proximal, mid, distal, chiasm, tract). Images were masked to include only the convex hull of V5-labeled tissue. The same ridge detection process used above was implemented, and the standard deviation of angles informed the cohesiveness of processes/angles. This analysis was done separately on the same tissue region corresponding to V5 and ß (iii) tubulin channels and standard deviation values for each channel were paired for a correlation analysis in Fig. 5. High standard deviation measurements signified that contours were more randomly oriented and lower deviation measurements showed a higher number of contours oriented in the same direction. Retinal astrocyte images were taken from our previously acquired datasets[12, 13].

Statistics were computed in GraphPad prism. If a test with underlying assumptions of normality was used, normality was assessed using the D’Agostino & Pearson test.

### Software

Software and versions used are as follows: Adobe Illustrator v29.7.1, GraphPad Prism v10.2.2, Python v3.10, Scikit Learn v1.2.2 [25], SymPy v1.14.0 [26], and Fiji ImageJ v2.35 [27].

## Results

### Astrocyte Morphology Varies with Location in the Optic Nerve

**Fig. 1:**
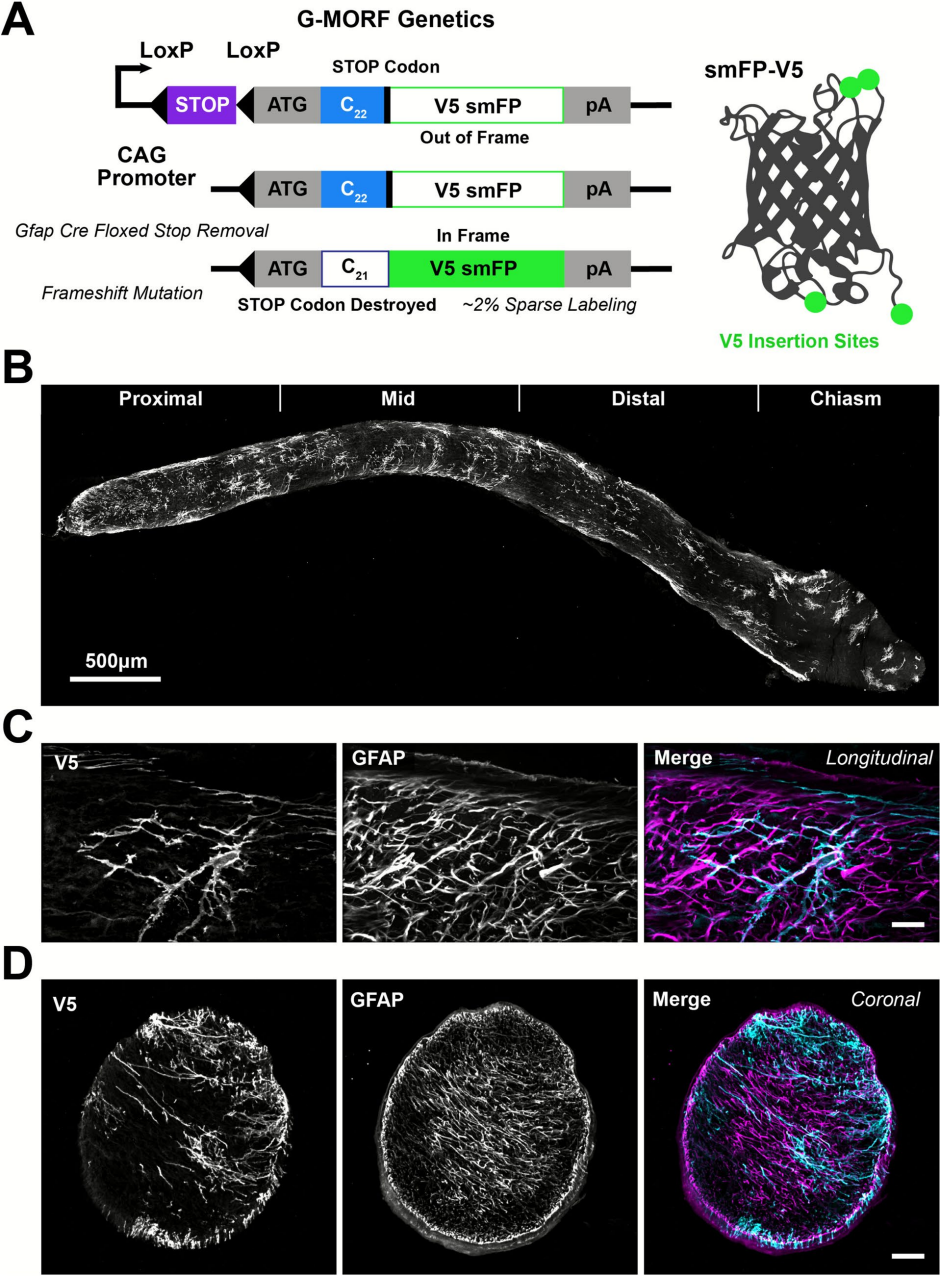
G-MORF mice exhibit sparse labeling of astrocytes in the optic nerve. **(A)** Genetics of G-MORF mice. Transcriptional STOP is removed with Gfap expression-dependent Cre recombinase, and translational STOP is removed with a stochastic frameshift mutation during development. The result is sparse, membrane-directed, expression of a V5-tagged spaghetti monster fluorescent protein (smFP). **(B)** Example optic nerve from a G-MORF mouse (MORF3 x Gfap-Cre). Labeling is against the V5 epitope tag and visualizes astrocytes. Astrocyte labeling is less dense in the chiasm compared to the rest of the nerve proper. **(C-D)** Sparse labeling of V5-smFP tagged G-MORF astrocytes compared to Gfap labeling in longitudinal **(C,** scale 20 µm**)** and coronal **(D**, Scale 50 µm**)** sections. **B-D** are cryo-sections

**Fig. 2:**
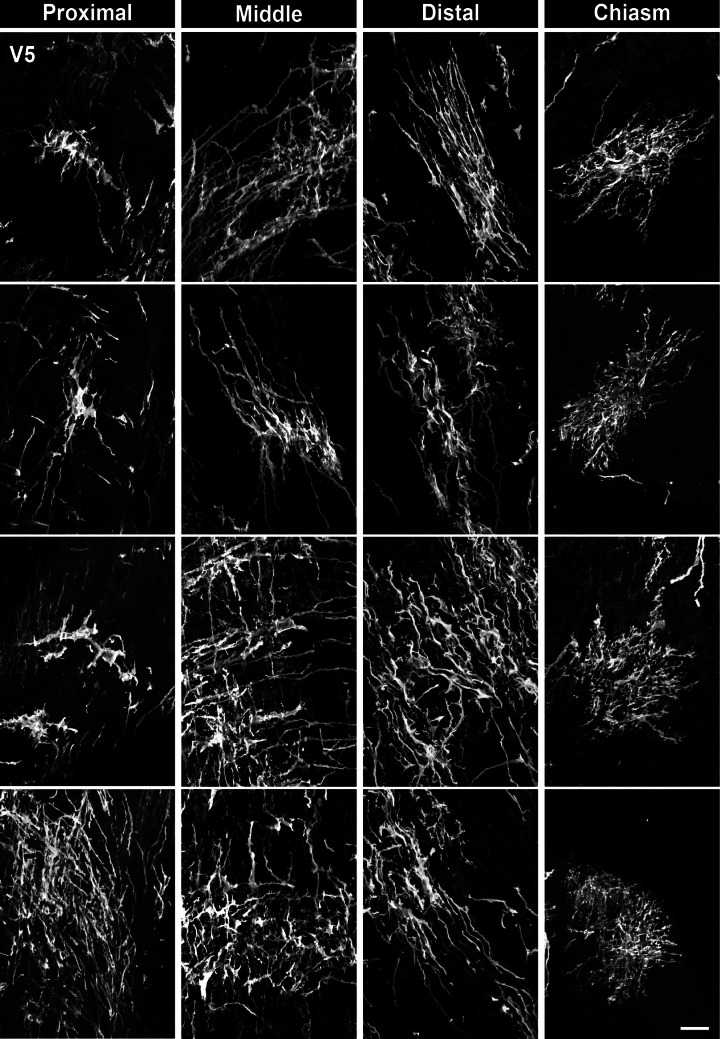
Astrocyte morphology varies with location in the optic nerve. Astrocyte morphology is heterogeneous in the optic nerve proper. Images are 60X confocal images of anti-V5-labeled G-MORF optic nerve cryo-sectioned tissue. The morphology is largely polarized in the same direction as axons. However, in the mid nerve, there is noticeably greater numbers of major astrocyte processes running perpendicular to the axons. Additionally, the starkest regional difference in morphology is between chiasm astrocytes and the rest of the nerve. In the chiasm, cells are bushier in appearance and less linearly polarized. Scale is 20 µm

Prior work has characterized astrocyte morphology in the optic nerve head in standalone studies as well as in comparison with the myelinated optic nerve proper as a whole[18, 20, 28, 29]. Extending this work to characterize morphology regionally within the myelinated optic nerve, we utilized the G-MORF mouse line which we created and used recently to characterize single-cell morphology in the retina[12–14]. The G-MORF mouse line stochastically expresses a membrane-directed, poly-V5 epitope tagged reporter protein in cells positive for glial fibrillary acidic protein (Gfap, Fig. 1A). While the reporter protein is a spaghetti monster fluorescent protein, its fluorescence capacity is broken upon the addition of V5 epitope tags (used for signal amplification in immunohistochemistry). In the following images, an anti-V5 antibody is used to reveal reporter protein expression in astrocyte plasma membrane. In the optic nerve, anti-V5 antibody sparsely labels astrocytes (Fig. 1B-D). While attempting to reconstruct the morphology of individual cells, we found that the density of labeled processes was much higher than in the retina, and that the ability to easily find well-separated cells was determined by the location in the nerve[12]. For example, we could easily find well-separated, labeled astrocytes in the optic chiasm whereas in the proximal nerve many adjacent cell processes were labeled (Fig. 2).

### The Orientation of Astrocyte Processes is a Function of Optic Nerve Position

While imaging longitudinal sections of optic nerve, we noticed that the orientation of labeled cells appeared to change along the longitudinal axis of the nerve (Fig. 1B). In the following paragraphs, the longitudinal axis of the nerve refers to the path from the eye to the chiasm- the long axis in longitudinal sections. The transverse axis refers to the short axis in a longitudinal section. The vertical axis refers to the direction perpendicular to both the longitudinal and transverse axes (vertical in coronal sections, Fig. S2).

Labeling of the V5-tagged reporter protein in longitudinal sections results in labeling of process fragments in the proximal nerve, often without clear view of the entire cell. This labeling pattern could arise if astrocytes were arranged with processes oriented in the direction of the vertical axis. In the mid nerve, full cells were observed with processes along the transverse axis as well as the longitudinal axis. This labeling could arise if cells were arranged roughly in the plane of the transverse and longitudinal axes. In the chiasm, full cells were also observed but were more compact in shape.

To test the idea that astrocyte morphology changes polarization along the length of the nerve, we took serial coronal sections through the entirety of the optic nerve at a step size of 15–25 µm. We labeled the sections for Gfap and segmented the resultant images for astrocyte process fragments. Because V5 expression is inherently stochastic, Gfap was chosen as a more consistent marker of astrocyte morphology to quantify along the length of the nerve. In the retina, the labeling of astrocytes in G-MORF mice is relatively uniform across the tissue, and cells are well-separated in a single plane of the Nerve Fiber Layer. This facilitates single-cell analysis. However, in the optic nerve, labeling is far less uniform. Additionally, because each coronal section of the nerve is thin (25 µm), the variability in amount of cells labeled per section is high (with some sections having no labeled cells). If V5-labeled cells were used in this analysis, the results would not be representative of the population of all astrocytes in the section, but rather just the few which were stochastically labeled. Additionally, because optic nerve astrocytes are more fibrous in appearance, Gfap labeling more faithfully shows the astrocyte volume than what could be expected in the retina.

Segmented Gfap process contours with an area less than 25µm^2^ were taken as longitudinal processes and those with larger area taken as transverse or vertical. Longitudinal fibers cut in coronal section will have a small cross-sectional area compared to transverse and vertically-oriented fibers. The ratio of longitudinal to transverse/vertical process area was collected for each position along the nerve. For each section, we also calculated the average angle made by transverse/vertical astrocyte processes and the transverse axis.

**Fig. 3:**
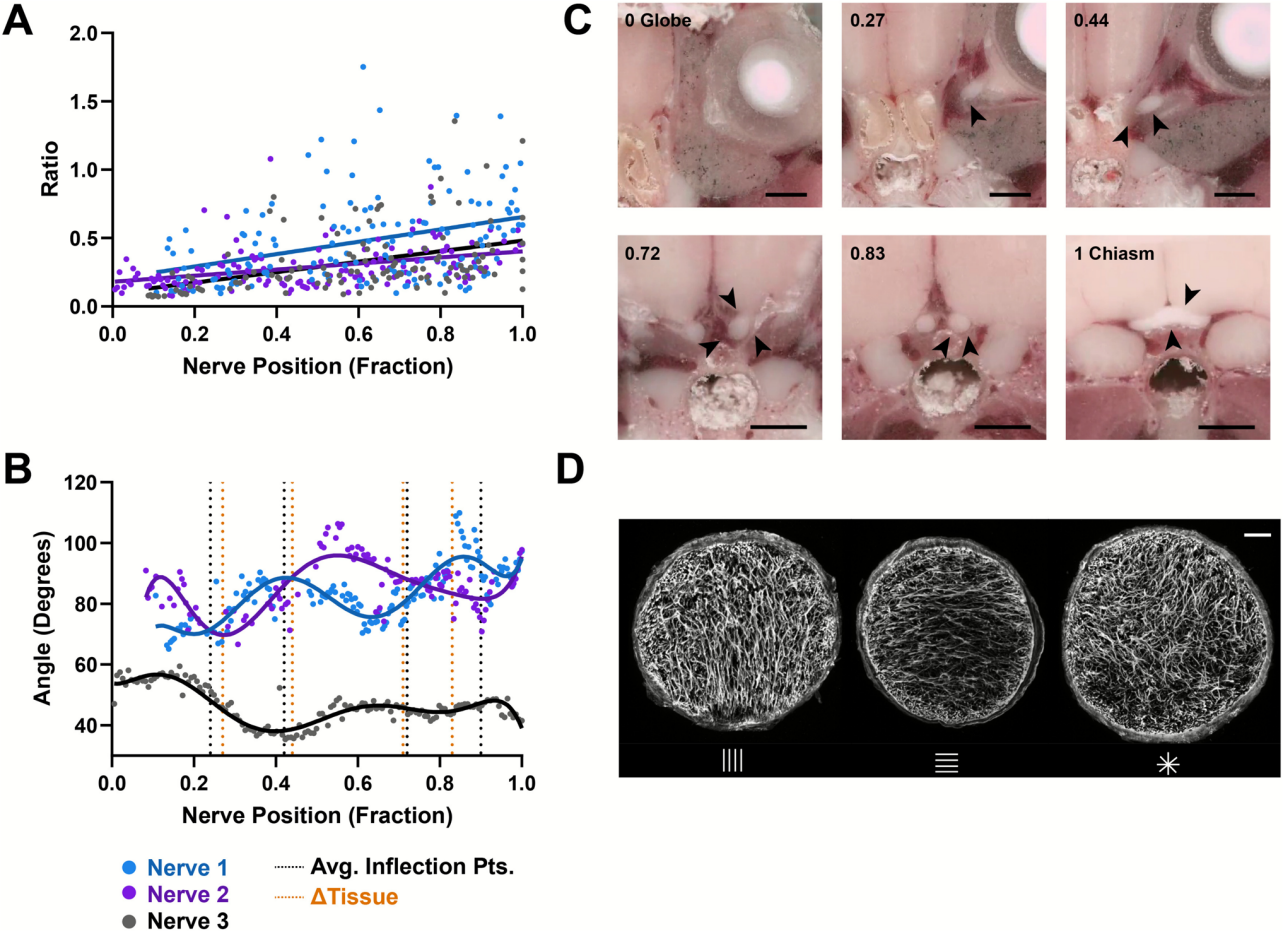
The orientation of astrocyte processes is a function of optic nerve position. **(A)** The percentage of longitudinal astrocyte fibers increases with distance along the optic nerve towards the chiasm. In coronal sections, particles recognized in binarized Gfap labeling of area < = 25µm^2^ were taken as longitudinal processes. Plotted is the ratio of longitudinal process area to transverse & vertical process area. **(B)** The average astrocyte process angle smoothly rotates about the longitudinal axis as a function of optic nerve position. Plotted is the average angle of contours (Gfap segments) along three individual nerves, found using ImageJ’s Ridge Analysis plugin with respect to horizontal, weighted by process length. Oscillatory patterns were fit with a 7th degree polynomial and the critical points closely follow changes in tissue environment of the optic nerve (vertical dotted lines). **(C)** Serial sections of whole mouse head adapted from Foo *et. al.*, [22]. Each image corresponds to a different local environment of the optic nerve (start, end, and Δtissue points in **B**), identified through arrows. Scale indicates 1 mm. **(D)** Representative coronal paraffin sections immunolabeled for Gfap showing different average angles of Gfap fragments (vertical, horizontal, mixed). Scale 50 µm. 3 nerves from 3 mice were analyzed. Analysis and images in **A-B,D** are from paraffin sections

Moving distally from the proximal nerve, the ratio of longitudinal to transverse/vertical processes increases (Fig. 3A, Fig. S2-S3A). Furthermore, the average angle of transverse/vertical Gfap fibers smoothly oscillates as a function of optic nerve position (Fig. 3B, Fig. S3B). We analyzed three full length optic nerves from three mice and found that while the exact pattern varies from nerve to nerve, likely due in part to initial orientation of the fixed nerve in the embedding, the observation of an oscillating angle in average astrocyte fiber orientation is consistent.

Nerves were analyzed from animals perfused with 4% paraformaldehyde instead of fresh, post-fixed samples. This ensures that we capture the state of the nerve as it exists *in vivo*. If a fresh nerve were used and straightened prior to fixation and sectioning, the observation of an oscillating fiber angle could be due to nerve torsion. Additionally, we ensured that this was not a sectioning artifact by tracking each section orientation relative to the embedding block (*see Methods, Fig. S1*). Because the initial orientation of each nerve in embedding changes slightly between samples, the maxima and minima of each plot do not perfectly align. Instead, it is the inflection points that are comparable between nerves. Additionally, to compensate for slight differences in nerve length that may arise between animals, total nerve length was normalized to a value of 1, with endpoints being easily identifiable proximal nerve (sclera attached) and the transition zone between chiasm and optic tract.

We found that each plot of Gfap process angle with respect to nerve position had 3–4 inflection points (that weren’t edge effects of the polynomial fit). When respective inflection point locations were averaged across all nerves, we noticed that the points closely corresponded to locations where the tissue environment surrounding the optic nerve changed (Fig. 3B-C). The average error between nerve environment change and inflection point location was $3.1 \pm 1.5$ % of nerve length. Using 10 µm thick cryosections through a mouse head adapted from supplemental data in a publication by *Foo et. al*, we traced the path of an optic nerve from the chiasm to the eye and noted regions where the local tissue environment changes- blind to any of the Gfap process polarization data (Fig. 3C)[22]. Local environment changes were determined visually, where tissue surrounding the optic nerve changed composition- tracing sections from the chiasm to the nerve head.

We first identified all major tissues surrounding the optic nerve and chiasm. This includes the hypothalamus and diagonal band nucleus of the brain, the ventral aspect of the presphenoid bone and the optic foramen, the trigeminal nerve, extraocular rectus muscles, lacrimal and Harderian glands, and sclera of the globe. This was accomplished using the Allen Brain Atlas, a whole-body mouse computed tomography (CT) scan atlas by IMAIOS (link in methods), and a variety of publications[23, 24]. We noted these tissues in coronal sections through the entirety of the mouse head for which the optic nerve and chiasm were visible. When a change in the combination of surrounding tissues occurred, we noted the location. The composition of each unique tissue environments were as follows (from posterior to anterior): (1) optic chiasm situated with superior hypothalamus, inferior presphenoid bone, and inferolateral trigeminal nerve, (2) separated optic nerves with each nerve contacting the diagonal band nucleus and olfactory tubercle superiorly and laterally and the presphenoid bone inferiorly, (3) optic nerve separated from the brain and entering the optic foramen, (4) optic nerve immediately encapsulated by exterior ocular rectus muscles in the orbital cavity and bordered by lateral aspect of sphenoid bone and intraocular lacrimal/Harderian glands, (5) the optic nerve surrounded by rectus muscles and lacrimal/Harderian glands but in the regions where there is separation of the muscles from bones of the orbit, and (6) the optic nerve reaches the sclera of the globe and remains surrounded by rectus muscles and lacrimal/Harderian glands. Images in *Foo et. al* were taken of adult mice 8–14 weeks in age. This conforms well to the ages of mice used in our study. The normalization of nerve length between our study and the *Foo et. al* study allow comparisons to be made between datasets. Images at these locations, as well as the proximal and chiasmic region of the nerve are shown in Fig. 3C. Distinct tissue environment changes include degree of contact with extraocular muscles, bone, brain, and fusion of each nerve into the chiasm region to contact axons of the fellow nerve. Figure 3D shows example coronal sections from a single nerve where Gfap fibers are predominantly oriented first vertically, then horizontally, and finally in a mixed orientation.

### Chiasm Astrocytes are Similar in Morphology to those in the Brain

In addition to mechanical forces and extracellular matrix cues, astrocyte morphology appears to be driven by the structures within their local environment[12, 30]. In the optic nerve, the most abundant features are the ganglion cell axons. If astrocyte morphology in the nerve is driven by nearby axons, we would expect that when axon orientation changes, so does the astrocyte morphology. At the optic chiasm, axons from the left eye cross to the right optic tract and vice versa. Because of this, at a given point in the chiasm, axons can be polarized in multiple directions. This contrasts with the rest of the nerve where all axons largely travel in the same direction.

**Fig. 4:**
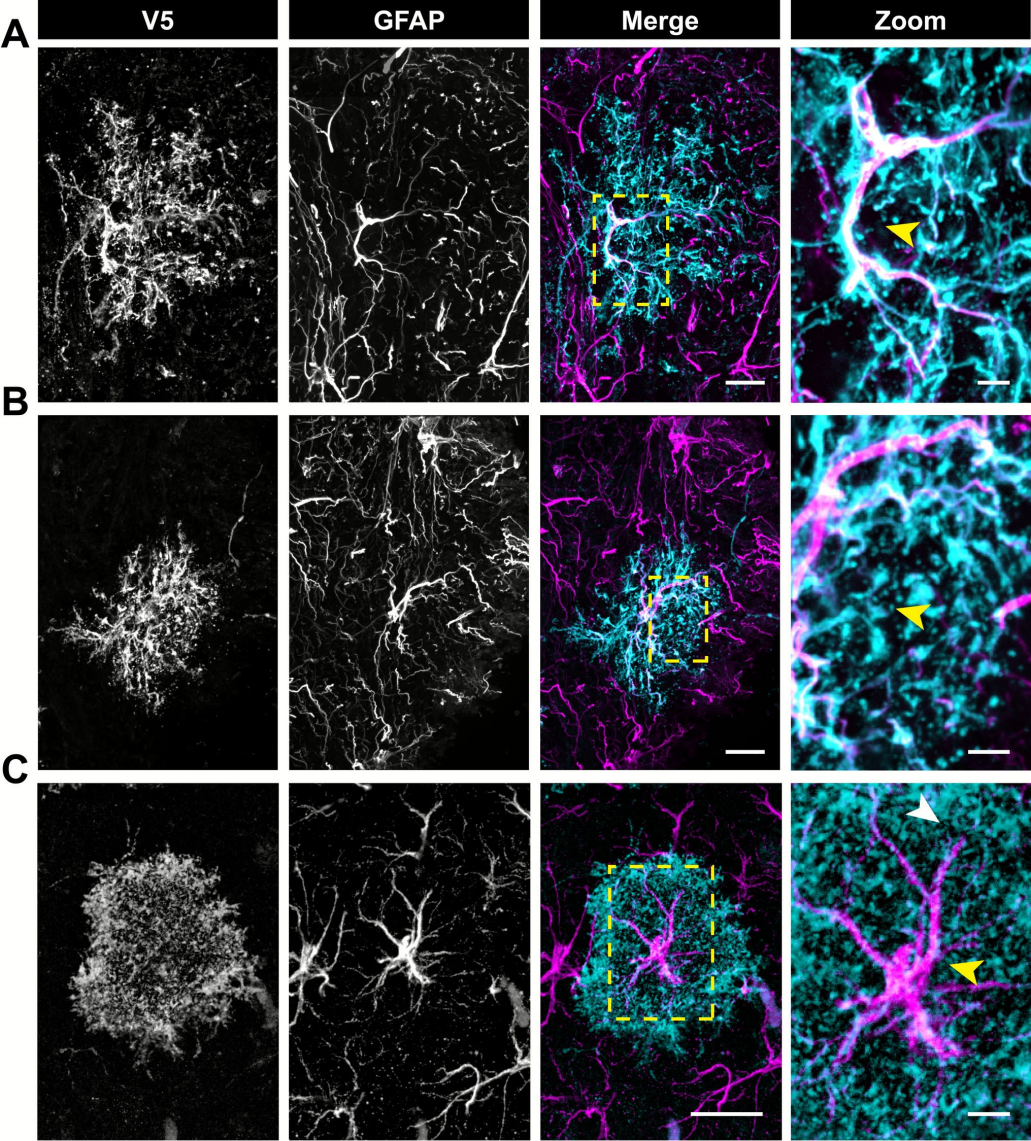
Chiasm astrocytes are similar in morphology to those in the brain. **(A-B)** Coronal sections of optic nerve chiasm labeled for V5 and Gfap. Cells typically have a central core with thick Gfap filaments (**A**, yellow arrow). Moving radially from this core is more expansive arborization with branches devoid of Gfap or expressed at low levels (**B**, yellow arrow). **(C)** Astrocyte in the hippocampus with radial morphology, a central core of thick Gfap filaments (yellow arrow) and a more extensive arborization with low levels or devoid of Gfap (white arrow). Scale 25 µm full size, 5 µm zoom. All tissue is cryo-sectioned

**Fig. 5:**
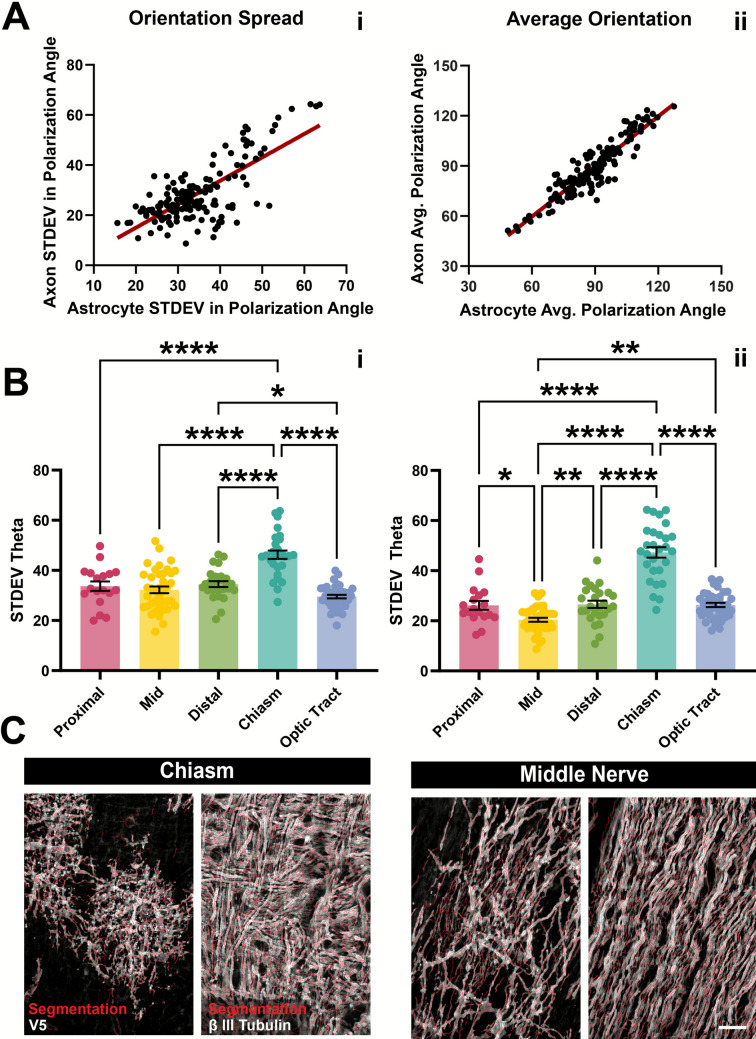
Astrocyte morphology is correlated with axon orientation. **(A-B)** The polarization of astrocytes is correlated with the polarization of axons in the optic nerve and optic tract (R^2^ = 0.5354, **A.i**). Average angle is similarly correlated (R^2^ = 0.8448, **A.ii**). Confocal images were segmented for astrocyte processes and axons. The standard deviation of the angles that these segments make with vertical is a measure of net cell polarization (closer to 0 has all processes going the same direction, higher values show segments in many directions). In the chiasm where axon fibers cross, astrocytes are bushier in appearance and have larger spread in segment angles **(B.i)** as do the axons **(B.ii). (C)** Example image segmentation of astrocyte processes (left) and their associated axons (right) in the chiasm and middle nerve. Scale is 20 µm. **** p < 0.0001; [axon angles] chiasm v. distal p < 0.0001; chiasm v. mid p < 0.0001, chiasm v. proximal p < 0.0001, chiasm v. optic tract p < 0.0001, distal v. mid p = 0.0086, mid v. proximal p = 0.0463, mid v. optic tract p = 0.0032 [astrocyte angles] chiasm v. distal p < 0.0001; chiasm v. mid p < 0.0001, chiasm v. proximal p < 0.0001, chiasm v. optic tract p < 0.0001, distal v. optic tract p = 0.0464. Statistics are ordinary one-way ANOVA with Tukey’s post-hoc test. Data pass D’Agostino & Pearson normality test. 153 images from 10 optic nerves of 6 mice were analyzed. Individual breakdown of images per group: Chiasm 28, Distal 26, Mid 39, Proximal 19, and Tract 41. Data and images correspond to cryo-sectioned tissue

**Fig. 6:**
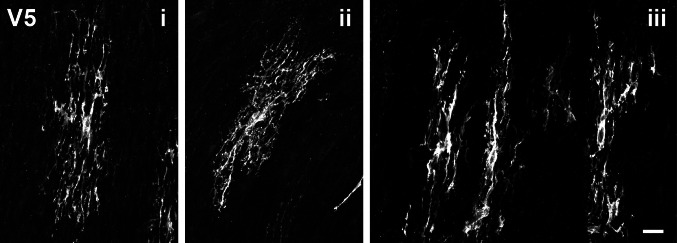
Optic Tract Astrocyte Morphology is Linearly Polarized.** (i-iii)** Images are 60X confocal images of V5-labeled G-MORF optic tract cryo-sectioned tissue. The morphology is largely polarized in the same direction as axons, and V5 labeling is relatively well-separated. Gross morphology is very similar to the optic nerve astrocytes but differs from those in the chiasm. Scale is 15 µm

In G-MORF optic nerves, we noticed a striking difference between astrocyte morphology in the chiasm vs. the rest of the nerve. In the chiasm, cells were more spherical or bushy in appearance compared to other regions (Fig. 4A-B). Interestingly, the morphology of chiasm astrocytes appears more similar to astrocytes in the brain gray matter than in the rest of the optic nerve or even the retina. In Fig. 4C we highlight an astrocyte in a G-MORF mouse hippocampus. The cell is radially polarized in a bushy overall morphology. Moreover, it has a strong central core of Gfap labeling which thins out along its extensive branching. Some processes appear negative in immunolabeling for Gfap. The same features are observed in chiasm astrocytes (Fig. 4A-B). In our previous work, we show that anti-V5 labeling in G-MORF animals is ground truth for astrocyte membrane morphology[12]. While Gfap is the most common astrocyte marker, it cannot reveal all fine process details. In Fig. 4, similarities should be drawn between chiasm astrocyte and gray matter astrocyte shape both in the core Gfap cytoskeleton and the full membranous morphology (anti-V5) which reveals a more compact and often spherical morphology not observed in the rest of the nerve (Fig. 2). The core region of Gfap corresponds to a less obvious volume in the V5 panels, largely because V5 reveals complete morphology so the cell core is less obvious. In Fig. 4A, more intense labeling of the core region by V5 is visible, and we hypothesize that this is because new protein is likely translated in the cell body and diffuses throughout the membrane from that point leading to higher intensity labeling at the cell core. We are unsure why this is not the case in all images. In longitudinal sections labeled for astrocyte reporter protein and axons, we segmented both astrocyte processes (anti-V5) and axons (ß III Tubulin, Fig. 5). ß (iii) tubulin is a neuron-specific isotype of ß tubulin and does not colocalize with astrocytes in the optic nerve (Fig. S4). Taking the standard deviation of the angles made by each segment with the longitudinal axis gives us a measure of the degree to which a cell is polarized in one direction (Fig. S3 B-C). If the spread of angles is low, all processes or axons project in the same direction. If the spread of angles is high, processes or axons are less cohesive in their projection direction. In the proximal, mid, and distal regions of the nerve, the spread of both astrocyte processes and axons were very similar (Fig. 5B). However, in the chiasm, the deviation was significantly higher (p < 0.0001). Moreover, both the average and standard deviation of axon angles was positively correlated with those of astrocyte process angles (Fig. 5). Moving past the chiasm and into the optic tract showed a reversion back to lower levels of both axon and astrocyte process angle spread. Astrocytes in the optic tract are more similar to astrocytes in the optic nerve than in the chiasm as well, highlighting that the morphology change is spatially transient and unique to the environment of the chiasm (Fig. 6).

### Optic Nerve Astrocytes Exhibit the Same Neuronal-Associated Morphological Motifs Found in the Retina

**Fig. 7:**
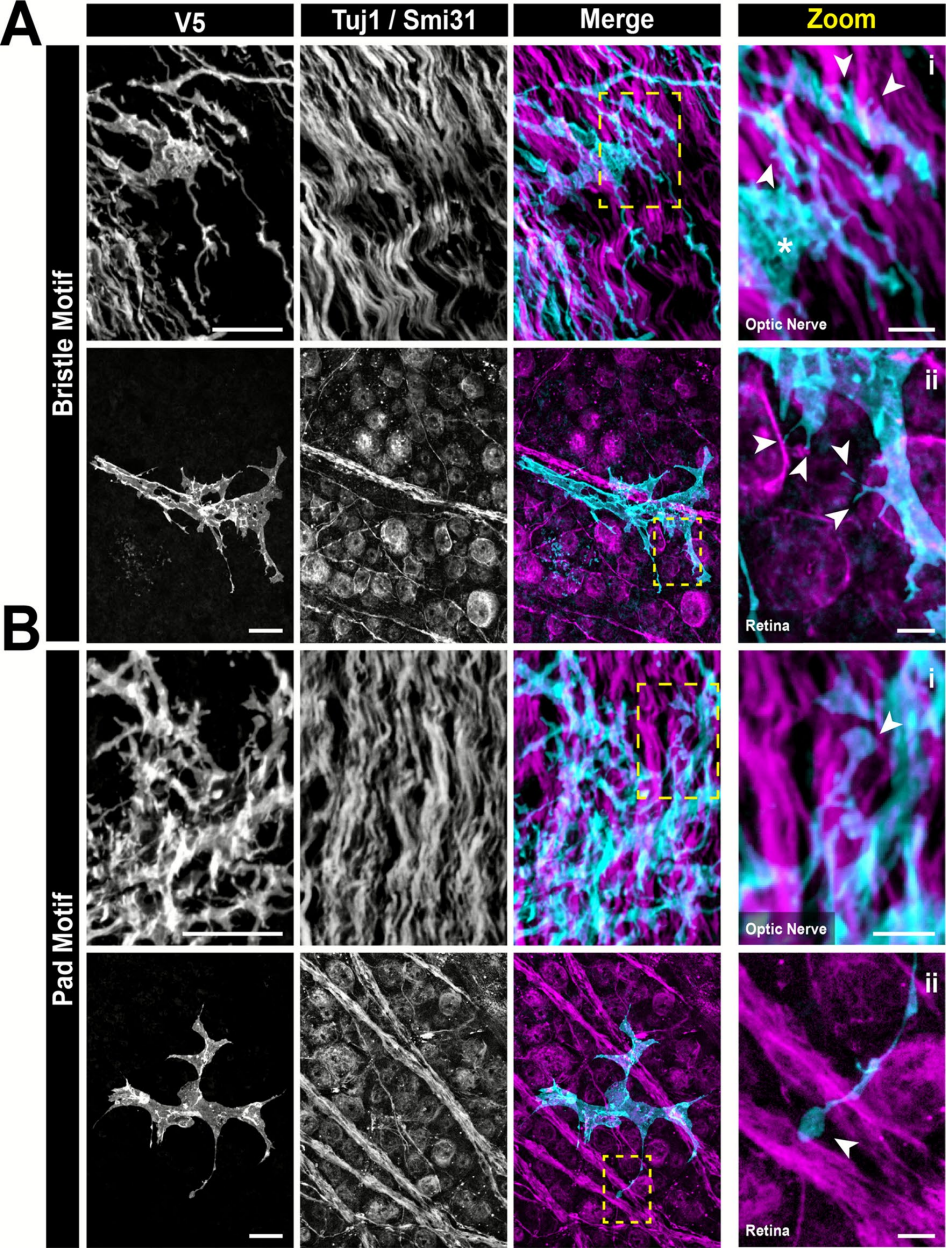
Optic nerve astrocytes exhibit the same neuronal-associated morphological motifs found in the retina. **(A)** Bristle and **(B)** pad motifs are found in both the **(i)** optic nerve and **(ii)** retina. Arrows indicate example motifs and yellow dashed boxes indicate the zoom region for the far-right panel. Scale bars indicate 20 µm and 5 µm for full-size and zoom images respectively. In **A**, the asterisk denotes a sail motif (See Fig. 8)

In our prior studies characterizing astrocyte morphology in the retina, we found recurring microstructures which were predictive of the underlying neuronal and vascular architecture of the inner retina[12]. We were interested to see if optic nerve astrocytes exhibit the same neuronal-associated motifs as their counterparts in the retina, and we report that they do. The most common and numerous motif in the optic nerve are bristles- short projections that contact axons (Fig. 7 A). We also observed the pad motif, a rounded projection ending which also contacts neuronal features (Fig. 7 B). In the retina, astrocytes resting on nerve fiber bundles have large regions of planar membrane called the sail motif. These motifs contact both axons and neuronal cell bodies in the retina. In the optic nerve, similar planar sheets of membrane were observed laminating between axon bundles as well (Fig. 7 asterisk and 8).

**Fig. 8:**
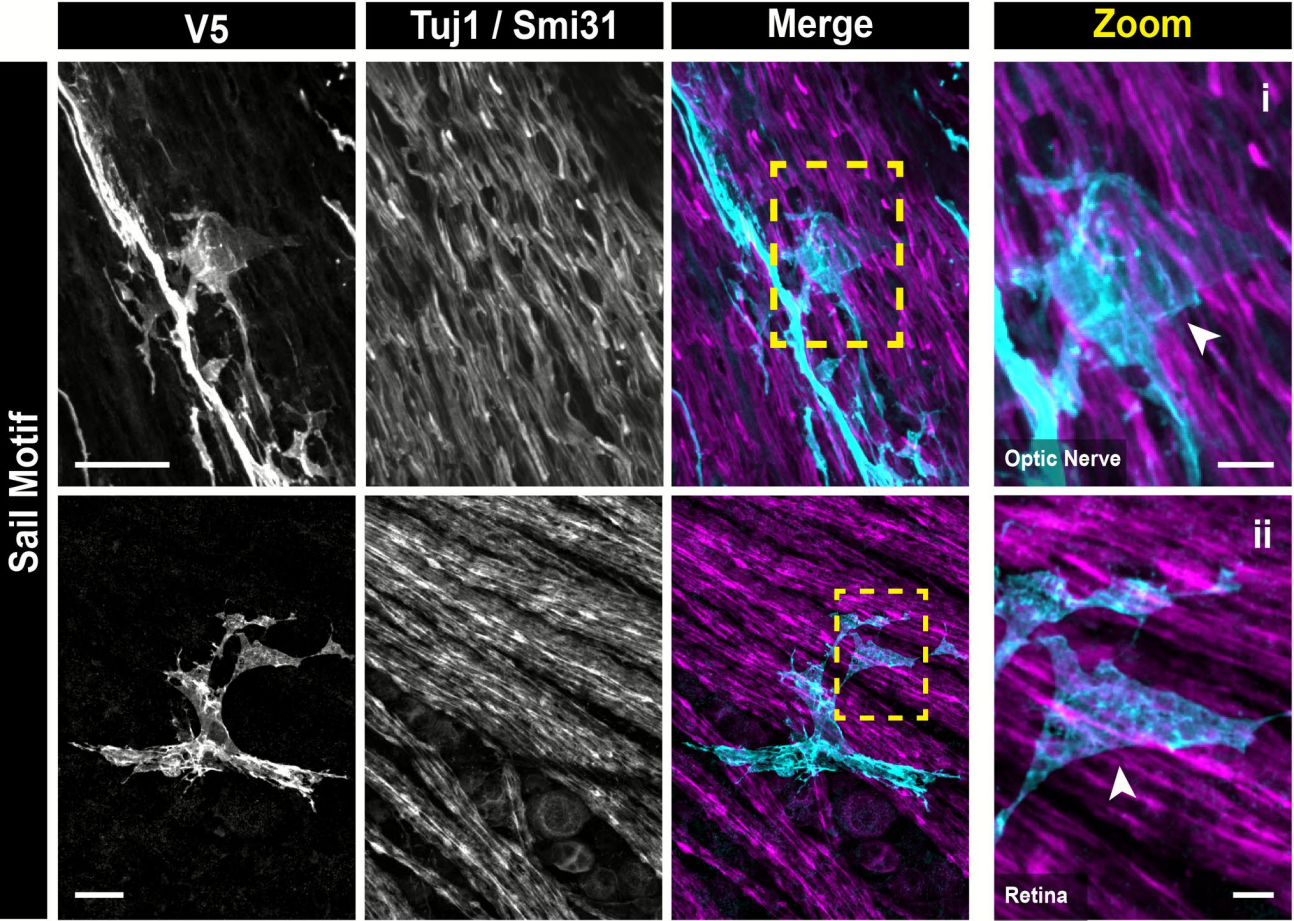
Optic nerve astrocytes exhibit the same neuronal-associated morphological motifs found in the retina. Sail motifs are found in both retinal and optic nerve astrocytes. Arrows indicate motif and yellow dashed boxes indicate the zoom region for the far-right panel. Scale bars indicate 20 μm and 5 μm for full-size and zoom images respectively

## Discussion

Recent studies in rodents show that the optic nerve has regional susceptibility to optic nerve crush and glaucomatous injury[8, 9, 31]. What drives regional susceptibility is a question of significant clinical value and the answer may result in eventual targeted therapies. It could be that regional susceptibility is due to (1) a distribution of transcriptionally unique cell types along the nerve or (2) a distribution of unique environments along the nerve, either in terms of physical surroundings or biochemical cues. Astrocytes could play a role in both possibilities, having demonstrated transcriptional heterogeneity in other regions of the CNS and an ability to respond to environmental forces[32–40]. Many studies have looked at astrocyte morphology in the naïve and glaucomatous optic nerve, but to our knowledge there has not been a systematic study of how that morphology changes as a function of optic nerve position. Moreover, studies either do not include the chiasm or fail to differentiate it as a specific region of interest. Because astrocytes play a significant role in tissue pathology, we sought to examine morphology regionally within the entire nerve, including the chiasm.

We find that the average orientation of astrocyte processes changes as a function of optic nerve position. A plot of these orientation angles reveals an oscillatory pattern with 3–4 transitions along the length of the nerve. While at any point along the nerve there are astrocyte processes oriented in many different directions, this shows that there is an orientation bias. Perhaps surprisingly, the transitions in process orientation closely align with transitions between tissue environments which surround the optic nerve. From chiasm towards the globe, we noted tissue environment changes (1) as the two nerves separate from the chiasm. The local environment changes from the nerve contacting brain superficially, fellow nerve medially and bone and connective tissue inferiorly and laterally to a state where connective tissue contacts the nerve medially and contacts brain and connective tissue laterally. (2) The next transition occurs as the nerve contacts the orbital bones laterally and pulls away from the skull inferiorly. A smaller proportion of the superior surface of the nerve contacts brain. Next, (3) the nerve rounds the orbital bones and is tightly encompassed by muscles which anchor to the nearby skull surface. Finally, (4) the nerve enters a region less compact in muscle and connective tissue before entering the globe.

To our knowledge, there is no known regional marker in the optic nerve that could be used to perfectly orient axes in coronal sections. While we tightly controlled nerve orientation throughout all steps in our processes, this opens the possibility that the oscillating pattern observed in Fig. 3 could arise due to (1) torsion of the nerve with otherwise uniform astrocyte morphology or (2) a sectioning artifact. Both of these possibilities are likely low in probability for a few reasons. With regards to the first point, from single axon tracing experiments, we know that the mouse optic nerve does not adopt a twist in vivo along the longitudinal axis[41]. If this were not the case, coronal sectioning of a nerve with otherwise uniform morphology *would* be expected to generate an oscillating pattern. However, even if this did occur in vivo, our use of a paraformaldehyde perfusion should lock the nerve in place as it is physiologically, so sectioning would reveal biologically relevant data. Additionally, from our longitudinal sections visualizing astrocyte morphology with V5 labeling, we observe distinct morphology in proximal, mid, distal, and chiasm regions of the nerve. If we expect to observe this trend in coronal sections, we would expect 3 transitions- roughly in line with our observations in Fig. 3. If morphology were constant but the single bend at the optic foramen were to induce an artifact in our quantification, we would at most expect a single step in values at that point along the nerve, but not a oscillating pattern.

With regards to the second point, sectioning (both paraffin and cryo-sections) routinely causes artifacts like delamination of tissue, shrinkage, etc[42]. Major artifacts like tissue tearing were rare but immediately recognizable in our own sections and were excluded from analysis. Furthermore, any tissue shrinkage would be expected to occur radially and thus be expected to affect all the data in a uniform way. Because our data were not biased to any one radial position in the coronal sections, we believe any shrinkage due to processing of paraffin tissue would not have a meaningful impact on our results. Lastly, if the tissue were cut at an unexpected angle, we would observe largely ellipsoid sections with crescent-shaped edges instead of roughly circular with clean edges as we see in our data. We attempted to combat both of these limitations with whole nerve clearing and light sheet microscopy. This would have had the added benefit of capturing whole cell morphology to add to our polarization data in Fig. 5; however, we had poor results with lipid clearing and antibody penetration (*data not shown*).

It is possible that the orientation of astrocyte processes informs us of the local forces exerted on the nerve. Perhaps more transversely oriented fibers are indicative of compressive forces along the nerve. We note an increase in transverse fibers near where the nerve curves about the orbital bones. The increase in transverse fibers may act as a spring, resisting compression as the nerve moves into the skull. Additionally, near this midpoint in the nerve, there may be compressive forces due to eye movements. As the eye rotates, the nerve is lengthened[43]. Because the nerve is anchored at the globe and the chiasm, this indicates that the central portion of the nerve is in compression. Moreover, if the skull itself acts as a fixed node, eye movements may induce additional strain midway between the globe and the skull (as a point that the nerve flexes about as the eye moves). Transcriptional data and immune labeling show that optic nerve astrocytes express Piezo mechanosensitive ion channels which astrocytes could use to sense these forces and alter their morphology[44]. This may be relevant in glaucoma where elevated intraocular pressure changes the force experienced at the optic nerve head, and remodeling of astrocytes in glaucomatous mouse optic nerves has been observed[45]. Both the changing environment and strain hypotheses are consistent with the data we present in this study.

In our prior work in the retina, we found that the local environment of the structures in the Nerve Fiber Layer and the Ganglion Cell layer appear to dictate the structure of the astrocytes[12–14, 46]. Recurring microstructures or motifs have characteristic interacting partners, be it neuronal cell bodies, axons, vascular structures or other glial cells. If this is true in a more general sense, we would expect astrocyte structure in the optic nerve to be driven primarily by the presence of axons which are the most abundant feature of the nerve. This hypothesis leads to the question whether astrocytes in the chiasm have a different morphology than those in the rest of the nerve because of the crossing of axons from one projection to the other. We find that this is indeed the case. Interestingly, astrocytes in the chiasm take on an appearance more akin to astrocytes in the gray matter of the brain than in the rest of the nerve. They appear more spherical or bushy in shape and have a central thick core of Gfap with thins out as the processes move further from the cell body. This morphology is spatially transient, being specific to the chiasm and is not prevalent in the rest of the optic nerve or the optic tract. In the brain, the dynamic orientation of axonal and dendritic structures in the tissue is more similar to the chiasm than in the nerve proper or certainly the retinal Nerve Fiber Layer. It could be that the multiple orientations of neuronal features in 3D space drives a more spherical morphology. By extension, the relatively planar distribution of axons in the retinal Nerve Fiber Layer and segregation of dendrites to the more distant Inner Plexiform Layer likely helps drive a flattened morphology of astrocytes in the Nerve Fiber Layer. Furthermore, we should expect that if neuronal features drive specific morphological motifs in retinal astrocytes, that the same motifs may be present in optic nerve astrocytes which also contact neuronal features. We find this to be true. Just like in the retina, optic nerve astrocytes express the neuronal associated bristle, pad, and sail motifs.

The changing of astrocyte morphology along the length of the nerve opens the possibility that there are distinct microenvironments along the length of the nerve which expose the cellular features there to unique forces and biochemical stimuli which could drive regional susceptibility to disease. Additionally, the discovery of the same microstructural motifs in nerve astrocytes as in the retina as well as observations of axon orientation correlation with astrocyte morphology in the nerve proper, chiasm, and optic tract provides additional evidence that the local environment plays a role in effecting astrocyte morphology. Future studies which are able to measure optic nerve strain or microenvironment in vivo will be critical in determining the relevance of these observations to disease.

## Supplementary Information

Below is the link to the electronic supplementary material.

ESM 1 (PNG 2.65 MB)

ESM 2 (PNG 281 KB)

ESM 3 (PNG 333 KB)

ESM 4 (PNG 6.02 MB)

## Data Availability

The authors will make available upon reasonable request all data leading to the conclusions reported in this paper.
