# Supplementary figures and images for: Regional Position and Axonal Environment Shape Astrocyte Morphology in the Mouse Optic Projection
Source: Mol Neurobiol. 2025 Nov 28;63(1):202. doi: 10.1007/s12035-025-05552-7 (PMC12662922; doi:10.1007/s12035-025-05552-7)

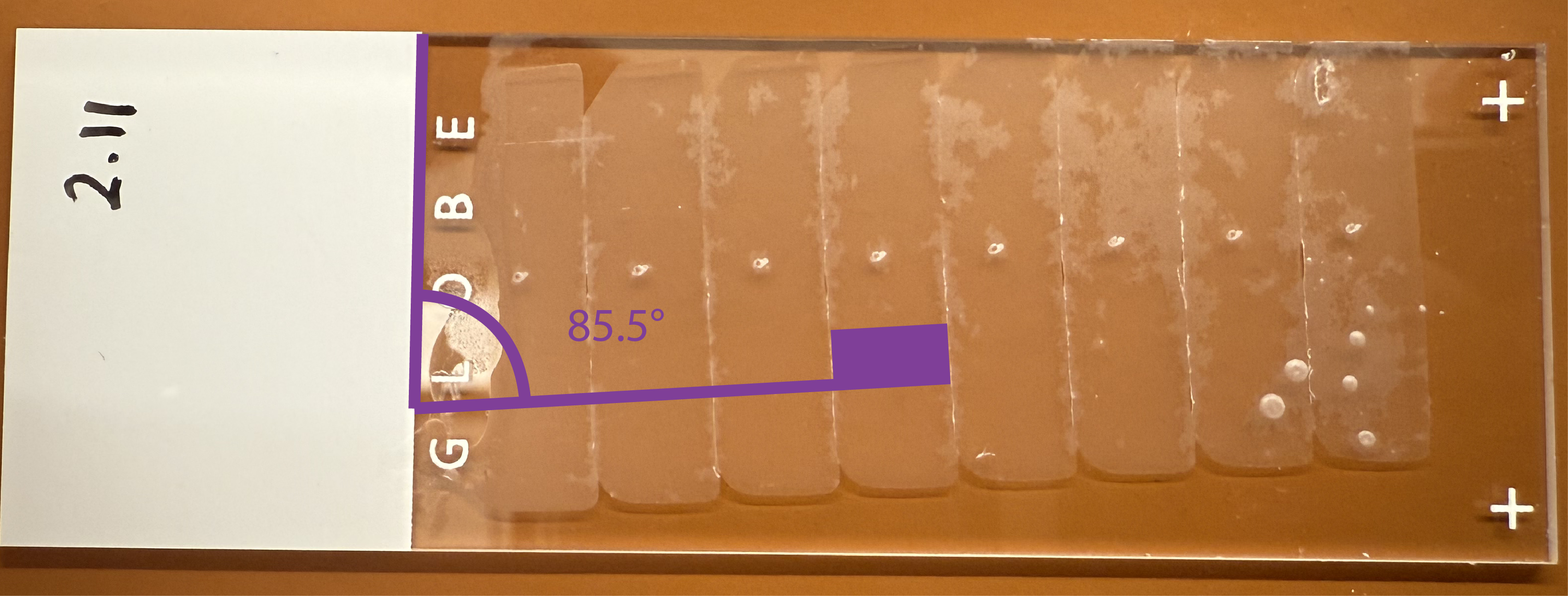

Supplement: Supplementary file 1 — (PNG 2.65 MB) [file 12035_2025_5552_MOESM1_ESM.png]

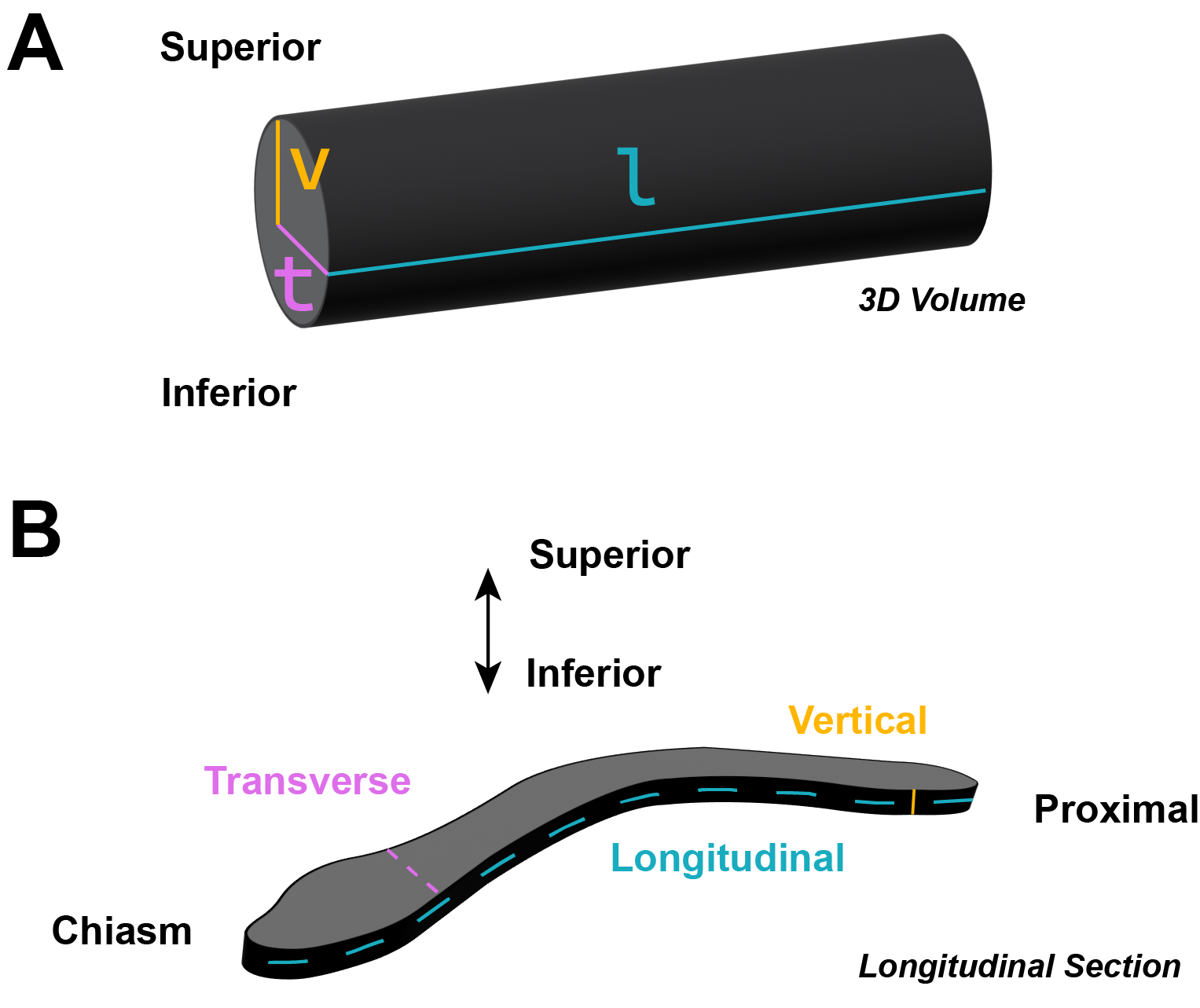

Supplement: Supplementary file 2 — (PNG 281 KB) [file 12035_2025_5552_MOESM2_ESM.png]

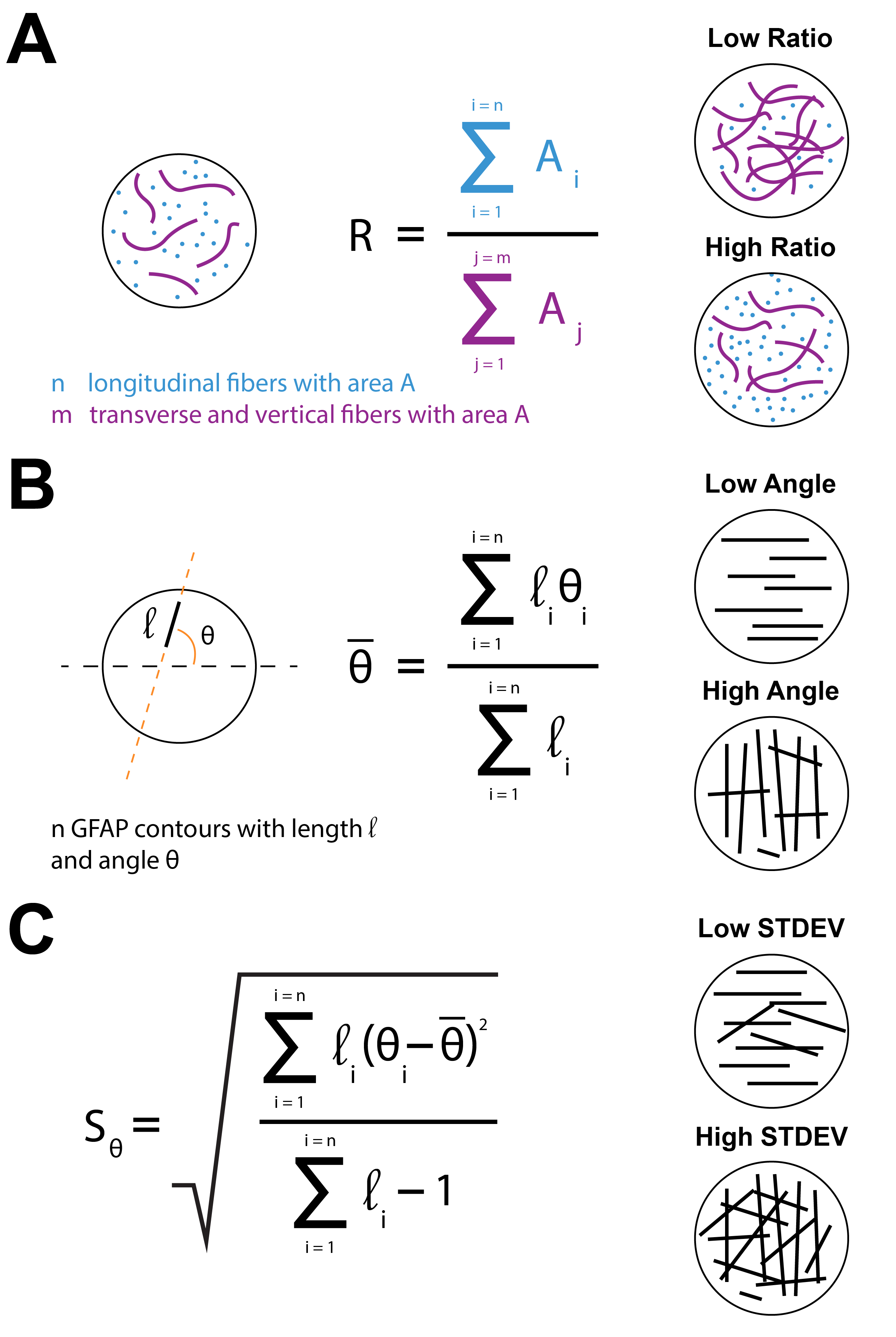

Supplement: Supplementary file 3 — (PNG 333 KB) [file 12035_2025_5552_MOESM3_ESM.png]

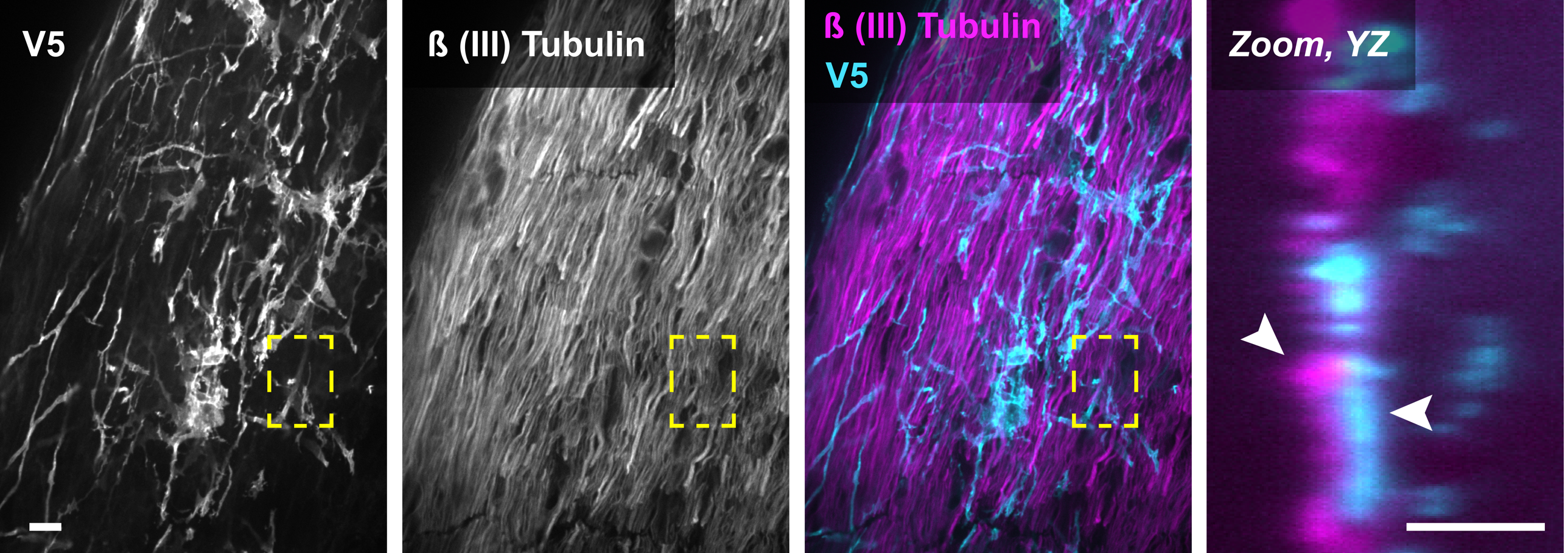

Supplement: Supplementary file 4 — (PNG 6.02 MB) [file 12035_2025_5552_MOESM4_ESM.png]
